# Transcriptional Regulation by Nuclear Corepressors and PGC-1****α****: Implications for Mitochondrial Quality Control and Insulin Sensitivity

**DOI:** 10.1155/2012/348245

**Published:** 2012-12-06

**Authors:** Zhengtang Qi, Shuzhe Ding

**Affiliations:** ^1^Key Laboratory of Adolescent Health Assessment and Exercise Intervention, East China Normal University, Shanghai 200241, China; ^2^College of Physical Education and Health, East China Normal University, Shanghai 200241, China

## Abstract

The peroxisome proliferator-activated receptors (PPARs) and estrogen-related receptor (ERR**α**) are ligand-activated nuclear receptors that coordinately regulate gene expression. Recent evidence suggests that nuclear corepressors, NCoR, RIP140, and SMRT, repress nuclear receptors-mediated transcriptional activity on specific promoters, and thus regulate insulin sensitivity, adipogenesis, mitochondrial number, and activity in vivo. Moreover, the coactivator PGC-1**α** that increases mitochondrial biogenesis during exercise and calorie restriction directly regulates autophagy in skeletal muscle and mitophagy in the pathogenesis of Parkinson's disease. In this paper, we discuss the PGC-1**α**'s novel role in mitochondrial quality control and the role of nuclear corepressors in regulating insulin sensitivity and interacting with PGC-1**α**.

## 1. Introduction

The transcriptional activity of nuclear receptors (NRs) is mediated by the recruitment of coactivators or corepressors to target genes. Transcriptional coregulators control the activity of many NRs and are thought to have wide-ranging effects on gene expression patterns. Of them, the best-studied group is the PPAR family. Peroxisome proliferator-activated receptor (PPAR) gamma coactivator 1 alpha (PGC-1*α*) is a central coactivator that mediates mitochondrial biogenesis, especially in skeletal muscle [1–3]. PGC-1*α* coactivates a broad range of transcription factors including PPARs, estrogen-related receptors (ERRs), nuclear respiratory factor 1 (NRF-1), myocyte enhancing factors (MEFs2), forkhead box O1 (FOXO1), and YY1. The ability of PGC-1*α* to increase the transcriptional activity of these and several other nuclear transcription factors provides the coactivator with the capacity to coordinate the large number of genes required for mitochondrial biogenesis. Nucleo-mitochondrial interactions depend on the interplay between transcription factors and members of the PGC-1 family (PGC-1*α*, PGC-1*β*, and PGC-1-related coactivator) [4]. In particular, PGC-1*α* interacts with NRF-1 and -2 to transactivate genes involved in the respiratory chain, mitochondrial import machinery and transcription factors of mtDNA (TFAM, TFB1M and TFB2M) [5]. Besides, transcriptional corepressors of NRs are known to regulate many genes, including a number of those that encode mitochondrial components and control insulin sensitivity [6, 7]. These studies highlight the essential role of NR corepressors and coactivators in maintaining mitochondrial homeostasis and describe an essential role for them in regulating insulin sensitivity.

Mitochondrial dysfunction plays an important and central role in the process of aging and the pathogenesis of many diseases, such as diabetes, cancer, obesity, cardiovascular disease, Alzheimer's disease, and Parkinson's disease. Skeletal muscle mitochondrial dysfunction is involved in the accumulation of intramyocellular lipid metabolites leading to lipotoxicity and insulin resistance [8]. Mitochondrial quality control (MQC) acts as a network of surveillance mechanisms including a wide range of relevant pathways that is important for the maintenance of mitochondrial population and integrity. In combination with mitochondrial biogenesis, the selective elimination of mitochondria by autophagy (i.e., mitophagy) regulates the changes in mitochondrial mass that are critical for the changes in metabolic needs. Nutrient starvation strongly induced autophagy that leads to bulk degradation of cytoplasmic components (proteins, organelles); so a large number of degradation products can be used to produce energy and components that are essential for cell survival in starvation conditions. In unstressed cells, autophagy is responsible for the replacement of long-lived proteins and organelles, because it may delete the exhausted, redundant, or unnecessary components. Thus, autophagy disorders lead to excessive accumulation of damaged cellular components, which may be involved in diabetes, neurodegenerative disorders, infectious diseases, and cancer [9]. Autophagy is also important for organelle function and insulin signaling loss of autophagy is a critical component of defective insulin action in obesity [10]. Both inhibition and alteration of autophagy can contribute to muscle disorders characterized by the accumulation of abnormal mitochondria [11]. Autophagy activity and expression of some key autophagy genes were suppressed in the presence of insulin resistance and hyperinsulinemia [12]. Hyperglycemia-associated oxidative stress induces autophagy, which may contribute to mitochondrial loss in soleus muscle of diabetic rats [13]. Mitochondrial dysfunction and oxidative stress mediate the impairment of insulin secretion in a mouse model where autophagy is inhibited by gene knockout within the pancreatic beta cell [14]. These results strongly suggest that the dysregulation of autophagy (mitophagy?) impairs mitochondrial homeostasis and thus leads to insulin resistance and metabolic disorders. In this paper, we discuss (1) the crosstalk between mitophagy and mitochondrial biogenesis and (2) the role of NR corepressors in regulating insulin action.

## 2. Coregulation of Mitophagy and Mitochondrial Biogenesis

The autophagy pathway can be induced and upregulated in response to intracellular reactive oxygen species (ROS) or extracellular oxidative stress. Thus, ROS play an important role in the activation of autophagy and are always involved in the process of autophagy survival or cell death that is initiated by starvation, pathogens, or death receptors [15]. Accumulating evidence indicates that p53 can modulate autophagy in a dual fashion, depending on its subcellular localization. Nuclear p53 transactivates proapoptotic, cell cyclearresting, and proautophagic genes that are able to promote autophagy, whereas cytoplasmic p53 can repress autophagy and promote apoptosis by translocation into mitochondria [16, 17]. Generally, AMP-activated protein kinase (AMPK) associates with, and directly phosphorylates, the Unc-51-like kinase (ULK1) and this modification is required for the induction of autophagy after glucose deprivation. When nutrients are plentiful, the mTORC1 complex phosphorylates ULK1, preventing its association and activation by AMPK [18]. So far, many authors have summarized the mechanisms that regulate autophagy and how they may contribute to cell survival and death. It is redundant to review the regulation of general autophagy in detail.

Mitophagy represents one type of selective autophagy during which whole mitochondria are engulfed by autophagic membranes and delivered to lysosomes leading to the formation of autolysosome. The process of mitophagy involves distinct steps to recognize defective or superfluous organelles and to target them to autophagosomes for degradation [19]. The ubiquitin ligase Parkin, ubiquitin, and p62 translocate to mitochondria and mediate the recognition of damaged mitochondria in preparing for mitophagy; this process is referred to as mitochondrial priming. Nix is a receptor on mitochondria, it can directly connect to the microtubule-associated protein 1 light chain 3 (LC3, Atg8) and gamma-aminobutyric acid type A receptor-associated protein (GABARAP); Atg8 and GABARAP are the component consisted in autophagy machinery. Nix also contributes to mitochondrial priming by controlling the mitochondrial translocation of Parkin [20]. In the initial step of Parkin-mediated mitophagy, PTEN-induced kinase 1 (PINK1) physically associates with Parkin so that they cooperatively recognize and label damaged mitochondria for mitophagy [21]. The ULK1/2 complex and an autophagy-related membrane protein Atg9A are recruited to depolarized mitochondria, they are required for further recruitment of downstream autophagy-related proteins (ATG) except LC3. At a later stage, LC3 is recruited and leads dysfunctional mitochondria into the autophagosome [22]. During the steps, identification of damaged mitochondria and induction of mitophagy are critical for selective mitochondrial autophagy. In mammals, loss of AMPK or ULK1 resulted in aberrant accumulation of p62 and defective mitophagy. ULK1 phosphorylation by AMPK is required for mitochondrial homeostasis and cell survival during starvation [23]. In muscular dystrophy, molecular markers of mitophagy (Parkin, PINK1, LC3, polyubiquitin, and p62) are localized to mitochondria. The genetic defect in choline kinase in muscle results in mitochondrial dysfunction and subsequent mitochondrial loss through the enhanced activation of mitophagy [24]. p53 and TP53-induced glycolysis and apoptosis regulator (TIGAR)-mediated inhibition of myocyte mitophagy are responsible for the impairment of mitochondrial integrity and apoptosis. The activation of Bcl-2/adenovirus E1B 19 kDa-interacting protein 3 (Bnip3) and mitophagy due to p53/TIGAR knockout (KO) are reversed with antioxidant N-acetyl-cysteine, indicating that this adaptive response requires ROS signal [25]. Together, autophagy-dependent mitochondrial turnover in response to cellular stress is necessary for maintaining mitochondrial quality.

**Figure 1:**
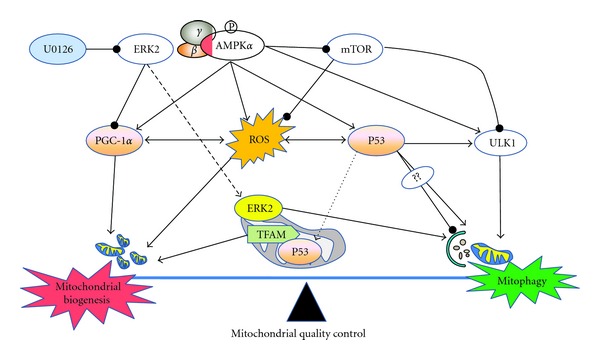
A well-controlled regulation of mitochondrial quality via mitochondrial biogenesis and mitophagy. Mitophagy, in conjunction with mitochondrial biogenesis, regulates the changes in mitochondrial number that are required to meet metabolic demand. Activated AMPK acutely triggers ULK1-dependent mitophagy and simultaneously triggers the biogenesis of new mitochondria via effects on PGC-1*α*-dependent transcription. Conversely, mTOR represses mitochondrial biogenesis and ULK1-dependent mitophagy when nutrients are plentiful. These dual processes controlled by AMPK and mTOR determine the net effect of replacing defective mitochondria with new functional mitochondria. AMPK: AMP-activated protein kinase; mTOR: mammalian target of rapamycin; PGC-1*α*: PPARgamma coactivator 1-alpha; ULK1: the mammalian Atg1 homologs, uncoordinated family member (unc)-51: like kinase 1; ERK2: the extracellular signal-regulated protein kinase 2; U0126: ERK inhibitor.

Mitochondrial biogenesis is enhanced in response to various physiological stimuli, like contractile activity, exposure to low temperatures, caloric restriction, and stem cells differentiation. Mitochondrial dysfunction may initiate a retrograde response, enabling cell adaptation through increased mitochondrial biogenesis [26] and also increase the elimination of dysfunctional mitochondria with mitophagy [27]. Interestingly, AMPK both triggers the acute destruction of defective mitochondria through a ULK1-dependent stimulation of mitophagy, and stimulates mitochondrial biogenesis through PGC-1*α*-dependent transcription [28]. Mammalian target of rapamycin (mTOR) signaling maintains quiescence and function of hematopoietic stem cells (HSCs) by repressing mitochondrial biogenesis and ROS [29]. mTOR complex 1 (mTORC1) activity is not integral for the increase in mitochondrial content elicited by chronic contractile activity, but is required to maintain mitochondrial function and homeostasis in resting muscle [30]. mTORC1 promotes the expression of nuclear genes encoding mitochondrial proteins (NUGEMPs) in resting muscle cells via the interaction of the mTORC1 components, mTOR and raptor, the transcription factor YY1, and PGC-1*α* [31]. On the other hand, p53 that controls autophagy contributes significantly to the regulation of mitochondrial content. Mice without p53 have reduced endurance capacity and muscle performance [32]. p53 interacts with mitochondrial transcription factor A (TFAM) and regulates mitochondrial DNA (mtDNA) content. Overexpression of p53 in mouse myoblasts increases both TFAM and mtDNA levels [33]. p53 null mouse and p53 knockdown human primary fibroblasts exhibit mtDNA depletion and decreased mitochondrial mass. This is accompanied by a reduction of TFAM at the protein level. p53-depleted cells exhibit a significant disruption of cellular ROS homeostasis [34]. Briefly, p53 functions as mitocheckpoint protein and regulates mtDNA copy number and mitochondrial biogenesis [35]. It is noteworthy that similar regulators including ROS, p53, AMPK, and mTOR control autophagy, mitophagy and mitochondrial biogenesis (Figure 1). These regulators may serve as a node that integrates mitochondrial remodeling in response to external stimuli.

In addition, extracellular signal-regulated protein kinases (ERK) regulates both mitophagy and mitochondrial biogenesis. Mitochondrial localization of ERK2 activity regulates mitophagy and autophagic cell stress, the level of mitophagy was tightly correlated with ERK activity [36]. Inhibiting autophagy and mitophagy only partially restored mitochondrial content. In contrast, inhibiting activation of ERK1/2 conferred complete cytoprotection with full restoration of mitochondrial functional and morphological parameters [37]. This synergistic effect of ERK signalling is mediated by the transcription factor ERR*α* [38]. However, ERK inhibitor U0126 activates PGC-1*α*, NRF-1, and TFAM and attenuates memory deficits in Abeta-injected rats [39]. Thereby, PGC-1*α* and ERR*α* will be further expected to regulate insulin sensitivity by MAPK/ERK signalling [40], for its ability to coordinate mitochondrial biogenesis and mitophagy. Herein, what interests us is the crosstalk between mitophagy and mitochondrial biogenesis triggered by external stimuli, which defines both cellular and mitochondrial quality control programs.

## 3. Cross-Talk between Autophagy and Mitochondrial Biogenesis Triggered by Exercise and CR

It has been known for more than 40 years that exercise causes increases in skeletal muscle mitochondrial biogenesis. Increasing evidence suggests that AMPK/mTOR signalling mediates the process of exercise-induced mitochondrial biogenesis and muscle growth. The induction of autophagy in skeletal muscle after-exercise is able to prevent the accumulation of damaged organelles and maintain cellular homeostasis. Thus, proper activation of autophagy is required for muscle homeostasis during physical exercise [41–43]. Exercise-induced autophagy is required for muscle glucose homeostasis and protection against high-fat diet- induced glucose intolerance [44]. Exercise increased AMPK phosphorylation, which stimulates autophagy via the suppression of mTOR phosphorylation, immediately after exercise [45]. During endurance exercise, AMPK triggered a coordinated activation of autophagy, ubiquitin-proteasome pathway and mitochondrial remodeling including mitophagy [46]. Following an acute bout of resistance exercise, the rate of protein synthesis increases proportionally with the increase in protein degradation (autophagy?). The class III PI3K (phosphoinositide 3-kinase) Vps34 (vacuolar protein sorting mutant 34) controls both autophagy and amino acid signalling to mTOR and its downstream target p70S6K1. mVps34 (mammalian Vps34) could act as an internal amino acid sensor to mTOR after resistance exercise [47]. Exercise may promote both autophagy and mitochondrial biogenesis via AMPK/mTOR signalling, conferring positive impacts on skeletal muscle contractile and metabolic functions, such as increased mtDNA content, fatty acid oxidation, and muscle force. However, exercise improved muscle mass in diabetes-induced skeletal muscle atrophy by suppressing autophagy, suggesting the activation of autophagy in diabetes contributes to muscle atrophy [48]. Doxorubicin (DOX) administration increases the expression of autophagy genes in skeletal muscle, exercise can protect skeletal muscle against DOX-induced activation of autophagy [49]. These findings suggest that exercise does not always activate autophagy for renewal of cellular components, it can also decrease autophagy in autophagy-induced pathological conditions.

Calorie restriction (CR) increases muscle mitochondrial biogenesis in healthy humans. CR increased expression of genes-encoding proteins involved in mitochondrial function such as PGC-1*α*, TFAM, endothelial NO synthase (eNOS), and Sirtuin 1 (SIRT1). In parallel, mtDNA content was increased by CR [50]. Also, CR can induce a PGC-1*α*-dependent increase in mitochondria capable of efficient and balanced bioenergetics to reduce oxidative stress and attenuate age-dependent endogenous oxidative damage [51]. Although PGC-1*α* is a major regulator of the mitochondrial response to CR in skeletal muscle, neither PGC-1*α* nor mitochondrial biogenesis in skeletal muscle is required for the whole-body metabolic benefits of CR [52]. However, Hancock, et al. demonstrate that 30% CR does not induce an increase in mitochondria in heart, brain, liver, adipose tissue, or skeletal muscle in laboratory rodents. With the exception of long-chain acyl-CoA dehydrogenase protein level, which was increased approximately 60% in adipose tissue, none of the mitochondrial proteins or mRNAs were increased in rats subjected to 30% CR for 14 wk [53]. On the other hand, short-term fasting induces a dramatic upregulation in neuronal autophagy. The increased neuronal autophagy is revealed by increases in autophagosome abundance and characteristics and by diminished mTOR activity in vivo, demonstrated by a reduction in the levels of phosphorylated S6 ribosomal protein in Purkinje cells [54]. Mild CR can attenuate the age-related decline in autophagy in rodent skeletal muscle, which might be one of the mechanisms by which CR attenuates age-related cellular damage and cell death in skeletal muscle in vivo [55]. Long-term CR in mice promoted increased Sirt1 expression in aged kidney and attenuated hypoxia-associated mitochondrial and renal damage by enhancing Bnip3-dependent autophagy [56]. On the contrary, another study suggests that long-term CR reduces mitochondrial biogenesis and mitophagy are reduced [57]. These findings contradict the theory that CR increases mitochondrial protein turnover.

Anyway, mitochondrial biogenesis must be balanced by mitophagy, mitochondrial mass and function must be adapted to cellular energy demands and particular physiological conditions. Like CR, exercise can increase mitochondrial biogenesis and autophagy, but the beneficial effects of exercise and CR in vivo are not always demonstrated by activation of autophagy and mitochondrial biogenesis, in effect, exercise and CR are able to fine-tune the rate of autophagy and mitochondrial biogenesis according to cellular energy demands and pathological process. Understanding the beneficial mitochondrial changes conferred by CR and exercise will aid the design of therapies for mitochondria -related diseases.

## 4. PGC-1***α***: More than a Coactivator for Mitochondrial Biogenesis

**Figure 2:**
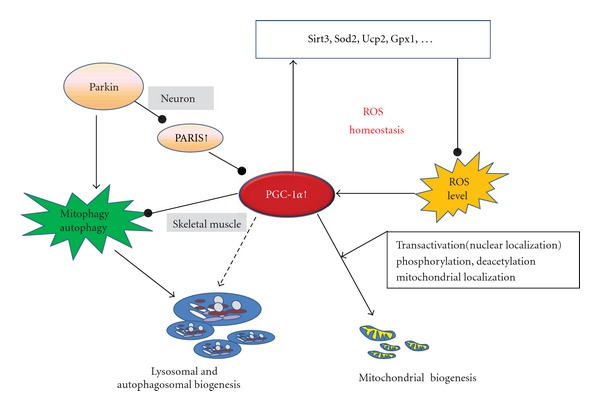
A central role of PGC-1*α* in coordinating autophagy and mitochondrial biogenesis. PGC-1*α* is a broad and powerful regulator of ROS metabolism and plays a dual role in skeletal muscle by activating autophagosomal biogenesis and preventing the induction of autophagy. Inhibited mitophagy impairs mitochondrial biogenesis via PGC-1*α* in neurodegeneration due to Parkin inactivation. PGC-1*α*: PPARgamma coactivator 1-alpha; Parkin: an E3 ubiquitin ligase that mediates the ubiquitination of protein substrates in mitophagy induction; PARIS, a zinc finger protein that accumulates in models of Parkin inactivation and in human Parkinson disease.

Mitochondrial biogenesis is determined by well-regulated PGC-1*α*, including the transcriptional regulation, the fine-tuning of its final activity via posttranslational modifications, and regional actions via its subcellular localization (Figure 2). Posttranslational modifications of PGC-1*α* by the deacetylase SIRT1 and the kinase AMPK are involved in exercise-induced mitochondrial biogenesis in skeletal muscle. SIRT1 deactetylation is proposed as a potential activator of PGC-1*α* transcriptional activity [58]. The direct phosphorylations of the PGC-1*α* by AMPK at Thr-177 and Ser-538 are required for the PGC-1*α*-dependent induction of the PGC-1*α* promoter [59]. Chronic exercise induces mitochondrial biogenesis in wild-type mice, which may require intact AMPK activation and involve SIRT1-dependent PGC-1*α* deacetylation [60]. Recently, PGC-1*α* has been shown to reside in mitochondria, where PGC-1*α* is in a complex with TFAM at mtDNA D-loop. This interaction was increased by exercise, similar to the increased binding of PGC-1*α* at the* Nrf1 *promoter. In response to exercise, PGC-1*α* relocalizes into nuclear and mitochondrial compartments where it may facilitate nuclear-mitochondrial DNA cross-talk to promote net mitochondrial biogenesis [61].

Exercise that initiates mitochondrial biogenesis and PGC-1*α* signaling also induces autophagy. Mitochondrial biogenesis and autophagy are both vital for cell survival; PGC-1*α* has been widely involved in cellular responses to stressors. Connections between ROS and autophagy are observed in diverse pathological conditions; recent advances in the field of redox regulation of autophagy focus on the role of mitochondria as a source of ROS and on mitophagy as a means for the clearance of ROS [15]. The net effect of AMPK activation on PGC-1*α* expression was a result of increased transcriptional activation, counterbalanced by reduced ROS production [62]. *Sirt 3* functions as a downstream target gene of PGC-1*α* and mediates the PGC-1*α* effects on ROS production [63]. In neuron, PGC-1*α* is a master regulator of ROS scavenging enzymes including manganese superoxide dismutase (MnSOD) and the uncoupling protein 2 (UCP2), both are mitochondrial proteins and may contribute to neuronal survival [64]. PGC-1*α* is required for the induction of many ROS-detoxifying enzymes, including glutathione peroxidase (GPX1) and MnSOD. Increasing PGC-1*α* levels dramatically protects neural cells in culture from oxidative-stressor-mediated death [65]. These studies reveal that PGC-1*α* is a broad and powerful regulator of ROS metabolism, providing a potential pathway for the manipulation of autophagy (Figure 2).

Further, PGC-1*α* may regulate autophagy directly. Fiber type conversion by PGC-1*α* activates lysosomal and autophagosomal biogenesis in skeletal muscle, suggesting the role of PGC-1*α* as a regulator for organelle biogenesis—not only for mitochondria but also for lysosomes and autophagosomes [66]. However, increased PGC-1*α* levels in skeletal muscle during aging prevented muscle wasting by reducing apoptosis, autophagy, and proteasome degradation [67]. Elevated PGC-1*α* or PGC-1*β* prevented the induction of autophagy and atrophy-specific ubiquitin ligases by a constitutively active FOXO3 [68]. These results suggest a dual role of PGC-1*α* in the induction of skeletal muscle autophagy (Figure 2). It remains unclear how PGC-1*α* switches on or off the induction of autophagy to maintain muscle mass by means of transcriptional control. In addition, a combination of reduced mitochondrial biogenesis and increased mitophagy seems to be responsible for the decrease in mitochondrial content in diaphragm after hypoxia. Hypoxia decreased the content of PPAR*γ* and PGC-1*α*, whereas Bnip-3 was upregulated after hypoxia [69]. Exercise-induced fatigue and damage to muscle may be mediated via the regulation of mitochondrial dynamic remodeling, including the downregulation of PGC-1*α* and upregulation of autophagy [70]. In breast cancer cells, the protein kinase *Akt2* ablation initially resulted in an increase in the mitochondrial volume concomitantly with the upregulation of PGC-1*α*. Prolonged ablation of *Akt2* eventually led to cell death by the autophagy of the mitochondria (i.e., mitophagy) [71]. Overexpressing PGC-1*α* increased the abundance of OXPHOS protein complexes, conferred autophagy resistance under conditions of starvation and increased tumor growth by up to ~3-fold [72]. These results indicate that PGC-1*α* plays a role in regulating autophagy as well as mitochondrial biogenesis. Recent studies have established an impact of Parkin mutations on mitochondrial function and autophagy and suggested a potential involvement of the PGC-1*α* in the pathogenesis of Parkinson's disease (PD) [73]. PARIS is a zinc finger protein that accumulates in models of Parkin inactivation and in human PD brain. PARIS represses the expression of PGC-1*α* and the PGC-1*α* target gene, *Nrf1* by binding to insulin response sequences in the PGC-1*α* promoter. Moreover, overexpression of PARIS leads to the selective loss of dopamine (DA) neurons; this is reversed by either Parkin or PGC-1*α* coexpression [74]. The identification of PARIS provides a pathway by which inhibited mitophagy impairs mitochondrial biogenesis via PGC-1*α* in neurodegeneration due to Parkin inactivation (Figure 2). PGC-1*α* may be an important node of the multiple signaling pathways, enabling to integrate mitochondrial biogenesis and autophagy.

## 5. Nuclear Corepressors: Implications for Mitochondrial Activity and Insulin Sensitivity

NCoR1 is a corepressor of NRs, including PPAR*γ* in adipocytes and PPAR*δ* or ERR in myocytes. Ligand-dependent SUMOylation of the PPAR*γ* targets PPAR*γ* to NCoR1-HDAC3 (histone deacetylases 3) complexes on gene promoters. This in turn prevents recruitment of the ubiquitylation/19S proteosome machinery that mediates the removal of corepressor complexes [75] and autophagy induction [76]. For instance, transcriptional activation of many Toll-like receptors (TLRs) responsive genes requires an initial de-repression step, in which NCoR1 complexes are actively removed from the promoters of targets to relieve basal repression [77]. SUMO-PPAR*γ* inhibits NCoR1 clearance mechanisms, allowing promoter- and TLR-specific patterns of repression [78]. Apoptotic cells induce PPAR*γ* SUMOylation to attenuate the removal of NCoR1, thereby blocking transactivation of NF-kappaB [79]. In addition, PPAR*α* SUMOylation on lysine 185 downregulates its transcription activity through the selective recruitment of NCoR1 [80]. Generally, NCoR1 complexes are not cleared from the promoter and target genes in a repressed state. The dominant function of adipocyte NCoR1 is to transrepress PPAR*γ* and promote PPAR*γ* ser-273 phosphorylation by recruiting cyclin-dependent kinase 5 (Cdk5/CDK5); such that *Ncor1 *deletion leads to adipogenesis, reduced inflammation, and enhanced systemic insulin sensitivity [81]. Muscle-specific loss of NCoR1 in mice leads to an enhanced exercise endurance due to an increase of both muscle mass and of mitochondrial number and activity. The activation of selected transcription factors that control muscle function, such as MEF2, PPAR*δ*, and ERRs, underpins these phenotypic alterations [6].

**Figure 3:**
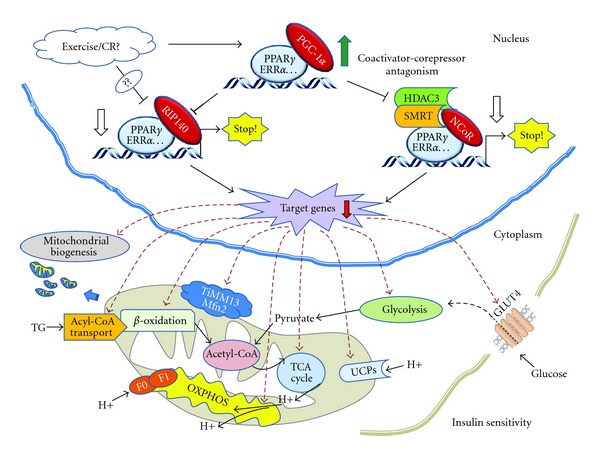
PGC-1*α*-NCoRs antagonism controls insulin sensitivity in metabolic tissues. In adipose and skeletal muscle, the transcriptional activity of PPAR*γ* and ERR*α* is responsible for the expression of gene networks that control glucose uptake, glycolysis, fatty acid oxidation, TCA cycle, OXPHOS, mitochondrial biogenesis, and uncoupling. Therefore, exercise and calorie restriction prevent obesity and insulin resistance probably by depressing NCoRs and increasing PGC-1*α*. PPAR*γ*, peroxisome proliferator-activated receptor gamma; PGC-1*α*: PPARgamma coactivator 1-alpha; ERR*α*, Estrogen-related receptor alpha; RIP140: the corepressor receptor-interacting protein 140; NCoR: nuclear corepressor; SMRT: silencing mediator of retinoid and thyroid hormone receptors; HDAC3: Histone deacetylases 3; TG: Triglyceride; UCPs: uncoupling proteins; Mfn2: mitofusin 2; TIMM13: mitochondrial import inner membrane translocase subunit Tim13; OXPHOS: oxidative phosphorylation.

The corepressor receptor-interacting protein 140 (RIP140) is also recruited by many NRs, including PPARs and ERRs. ERR*α* is capable of activating RIP140 gene transcription by two mechanisms, directly by binding to an estrogen receptor element/ERR element at −650/−633 and indirectly through *Sp1* binding sites in the proximal promoter. The upregulation of RIP140 by ERR*α* during adipogenesis provides an inhibitory feedback mechanism to control the expression of many NR target genes [82]. RIP140 is responsible for the suppression of gene networks that control catabolism in adipose tissue and skeletal muscle, including glucose uptake, glycolysis, tricarboxylic acid (TCA) cycle, fatty acid oxidation, mitochondrial biogenesis, OXPHOS, and mitochondrial uncoupling (Figure 3) [83]. Mice without RIP140 are lean with increased oxygen consumption and are resistant to high-fat diet-induced obesity and hepatic steatosis with an improved insulin sensitivity. Moreover, white adipocytes with targeted disruption of RIP140 express genes characteristic of brown fat including cell-death-inducing DFF45-like effector A (CIDEA), carnitine palmitoyl transferase-Ib (CPT1b), and the uncoupling protein 1 (UCP1) [84, 85]. Analysis of the *Ucp1* promoter showed RIP140 recruitment to a key enhancer element, demonstrating a direct role in repressing gene expression [86]. RIP140 requires ERR*α* to regulate hexose uptake and mitochondrial proteins succinate dehydrogenase subunit B and cytochrome oxidase Vb, although it likely acts through other NRs as well [87]. RIP140 is expressed in a fiber type-specific manner; manipulation of its levels in null, heterozygous, and transgenic mice demonstrate that low levels promote, while increased expression suppresses, the formation of oxidative fibers. Genes involved in fatty acid oxidation, OXPHOS, and mitochondrial biogenesis are upregulated in the absence of *RIP140*. The changes in expression are intrinsic to muscle cells and NR-regulated genes are direct targets for repression by RIP140 [88]. In the soleus, depletion of *RIP140* leads to increased glucose transporter 4 (GLUT4) trafficking and glucose uptake. AMPK phosphorylation/activity is increased. This is yet associated with increased *Ucp1 *expression and mitochondrial uncoupling [89]. These findings suggest the participation of RIP140 in the maintenance of energy homeostasis by acting as an inhibitor of energy production and particularly point to RIP140 as a promising therapeutic target in the treatment of insulin resistance. RIP140 is a major suppressor of adipocyte oxidative metabolism and mitochondrial biogenesis, as well as a negative regulator of whole-body glucose tolerance and energy expenditure.

Beyond as a nuclear corepressor, cytoplasmic RIP140 interacts with the Akt substrate AS160, thereby impeding AS160 phosphorylation by Akt; this in turn reduces GLUT4 trafficking [90]. Cytoplasmic RIP140 decreases adiponectin secretion [91]. Endothelin-1 promotes cytoplasmic accumulation of RIP140 through protein kinase C epsilon (PKC*ε*) pathway [92]. PKCepsilon stimulates arginine methylation of RIP140 for its nuclear-cytoplasmic export in an adipocyte differentiation. The methylated RIP140 recruits exportin 1 for nuclear export. As a result, the nuclear gene-repressive activity of RIP140 is reduced [93]. In adipocytes with high lipid contents, RIP140 increasingly accumulates in the cytoplasm and enhances triglyceride catabolism by directly interacting with perilipin, thus perilipin more efficiently recruits hormone-sensitive lipase (HSL) to lipid droplets and enhances adipose triglyceride lipase (ATGL) forming complex with CGI-58, an activator of ATGL [94]. Together, cytoplasmic RIP140 has been shown to play a role in the control of metabolism through direct regulation of lipolysis and glucose transport in adipocytes [95]. These studies highlight a new pathway for RIP140 to reduce glucose uptake and increase lipolysis.

As a nuclear receptor corepressor, silencing mediator of retinoid and thyroid hormone receptors (SMRT) is also recruited to PPAR*γ* via its C-terminal interacting domain, and the mutation of the proximal interacting domain does not interfere with recruitment via PPAR*γ* [96]. PPAR*γ* recruits SMRT and NCoR1 in the absence of ligand, these corepressors are capable of downregulating PPAR*γ*-mediated transcriptional activity. The PPAR*γ* ligand pioglitazone results in dissociation of the PPAR*γ*-corepressor complex [97]. Furthermore, estrogen receptors recruit SMRT and NCoR1 corepressors through contacts between the corepressor N terminus and the receptor DNA binding domain [98]. The core repression complex involves the recruitment of several proteins to a highly conserved repression domain within SMRT and NCoR1 [99]. HDAC3/NCoR1/SMRT axis is critical for maintaining chromatin structure and genomic stability [100]. SMRT stimulates the deacetylase activity of HDAC3 towards MEF2. Supporting the physical interaction and deacetylase activity, HDAC3 repressed MEF2-dependent transcription and inhibited myogenesis [101]. SMRT contains two interacting domains that mediate interactions with NRs. Mouse model termed SMRT (*mRID1*) in which targeted disruption of the first receptor interaction domain (*RID*) of SMRT disrupts interactions with a subset of NRs and leads to diet-induced superobesity associated with a depressed respiratory exchange ratio, decreased ambulatory activity, and insulin resistance. SMRT(*mRID1*) mice are both insulin-insensitive and refractory to the glucose-lowering effects of TZD (Thiazolidinediones) and AICAR (5-aminoimidazole-4-carboxamide 1-*β*-D-ribofuranoside). Lipid accumulation in brown adipose tissue was associated with reduced thermogenic capacity and mitochondrial biogenesis. Thus, SMRT promotes OXPHOS in adipose tissue and protects against diet-induced obesity and insulin resistance [7, 103]. SMRT expression and its occupancy on PPAR target gene promoters are increased with age in major metabolic tissues. Genetic manipulations to shift SMRT repression to *RID2*-associated NRs, notably PPARs, cause premature aging and related metabolic diseases accompanied by reduced mitochondrial function and antioxidant gene expression [102]. *SMRT *(*mRID1*) mice exhibit widespread metabolic defects including reduced respiration, altered insulin sensitivity, and 70% increased adiposity [103]. *SMRT* (+/−) mice develop an increased adiposity on a high-fat diet. Adipogenesis of mouse embryonic fibroblasts derived from these mice is increased. However, adipocyte insulin sensitivity is enhanced in *SMRT* (+/−) adipocytes [104]. These studies highlight the essential role of SMRT in maintaining a metabolic homeostasis and describe an essential role for SMRT in regulating adipogenesis and adipocyte insulin action.

Collectively, both NCoR and RIP140 may serve as a negative counterpart of PGC-1*α* by suppressing the transcriptional activity of NRs that control fatty acid oxidation, OXPHOS, and mitochondrial biogenesis in skeletal muscle and adipose; thus the deletion of them enhances insulin sensitivity and mitochondrial activity. However, SMRT that is required for adipocyte insulin sensitivity serves as an essential partner of NCoR when it is recruited by the NRs.

## 6. PGC-1***α*** Interacts with the Nuclear Corepressors

There are several studies suggesting that nuclear corepressor is a negative counterpart of PGC-1*α*, likely by blocking PGC-1*α* binding to the nuclear receptors. CIDEA, a mitochondrial protein, colocalizes around lipid droplets with perilipin, a regulator of lipolysis. CIDEA and other lipid droplet proteins define a novel, highly regulated pathway of triglyceride deposition in human white adipose [105]. The expression and promoter activity of CIDEA were repressed by RIP140 and induced by PGC-1*α*, mediated through the binding of ERR*α* and NRF-1 to their cognate binding sites. RIP140 interacts directly with PGC-1*α* and suppresses its activity. The direct antagonism of PGC-1*α* by RIP140 provides a mechanism for regulating target gene transcription via NR-dependent and -independent pathways [106]. Clenbuterol, a *β*2-adrenergic agonist, reciprocally alters PGC-1*α* and RIP140 and reduces fatty acid and pyruvate oxidation. In red and white muscles, clenbuterol induced reductions in mitochondrial content, proteins involved in fatty acid transport and oxidation, glucose transport, lactate transport monocarboxylate transporter, and pyruvate oxidation. These metabolic changes induced by clenbuterol were associated with reductions in PGC-1*α* and increases in RIP140 [107]. Further, SUMOylation of PGC-1*α* attenuates the transcriptional activity of the coactivator, probably by enhancing the interaction of PGC-1*α* with corepressor RIP140. Mutation that abolished the SUMOylation augments the activity of PGC-1*α* also in the context of PPAR*γ*-dependent transcription [108]. Overexpression of RIP140 can abrogate the PGC-1*α*-mediated induction of mitochondrial membrane potential elevation and mitochondrial biogenesis, and activate both autophagy and apoptosis pathways. RIP140 and PGC-1*α* exert antagonistic role in regulating cardiac energy state and mitochondrial biogenesis [109]. These results suggest that PGC-1*α* interacts with RIP140 by a competitive binding to the NRs and thus suppresses or enhances the transcription action of NRs. Unexpectedly, swim training increased markers of mitochondrial content in rat skeletal muscle independent of reductions in nuclear RIP140 protein. High-intensity exercise training in humans failed to reduce nuclear RIP140 content despite increasing skeletal muscle mitochondrial enzymes. An acute bout of exercise, AICAR treatment, and epinephrine injections increased the mRNA levels of PGC-1*α* independent of decreases in nuclear RIP140 protein [110]. Therefore, decreasing RIP140 is not required for exercise- and AICAR-mediated increases in skeletal muscle mitochondrial content.

In addition, heme binding to the nuclear heme receptor *Rev-erb* *α* recruits the NCoR/HDAC3 complex to repress the transcription of PGC-1*α*, a potent inducer of heme synthesis. Depletion of *Rev-erb *α** derepresses PGC-1*α*, resulting in increased heme levels. Conversely, increased *Rev-erb *α** reduces intracellular heme, and impairs mitochondrial respiration in a heme-dependent manner [111]. In primary cortical neurons, SMRT specifically antagonizes PGC-1*α*-mediated antioxidant effects. PGC-1*α* and SMRT are antagonistic regulators of neuronal vulnerability to oxidative stress. This coactivator-corepressor antagonism is regulated by the activity status of the cell, with implications for neuronal viability [112]. FK614, a novel PPAR*γ* modulator, dissociates NCoR and SMRT from PPAR*γ* as effectively as rosiglitazone and pioglitazone, but can also differentially induce a ligand specific interaction of PPAR*γ* with PGC-1*α* [113]. These findings suggest a potential antagonism between PGC-1*α* and nuclear corepressors. PPAR*γ* and ERR*α* activate the transcription of a broad range of genes that control glucose uptake, glycolysis, fatty acid oxidation, TCA cycle, OXPHOS, mitochondrial biogenesis, and uncoupling; thus, the treatments and drugs that can depress NCoRs activity or enhance PGC-1*α* activity are hoped to prevent obesity and insulin resistance (Figure 3).

## 7. Conclusion Remarks

Mitochondrial quality control depends upon a balance between biogenesis and autophagic destruction (mitophagy). A number of nuclear receptors control the induction of these processes by selectively recruiting transcription coactivators, such as PGC-1*α*, and corepressors such as NCoR and RIP140. The corepressors can block PGC-1*α* binding to the nuclear receptors, exercise and CR can increase it. A potential role for PPARs agonists in the adjuvant treatment of insulin resistance is advisable so far; thus enhancing PPARs transcription activity may increase mitochondrial biogenesis and glucose and fatty acid utilization. Recent studies reveal that PPARs transcription activity is repressed by nuclear corepressors. Further studies will have to provide a molecular understanding for an important role of PGC-1*α*-corepressors antagonism in mitochondrial quality control and a further deal with the possibility of depressing nuclear corepressors by exercise, CR, and drugs in the hope of preventing obesity and type 2 diabetes.
